# Supplementary material for: Meropenem Model-Informed Precision Dosing in the Treatment of Critically Ill Patients: Can We Use It?
Source: Antibiotics (Basel). 2023 Feb 13;12(2):383. doi: 10.3390/antibiotics12020383 (PMC9951903; doi:10.3390/antibiotics12020383)
Supplement: Supplementary file 1 [file antibiotics-12-00383-s001.zip › antibiotics-2197297-supplementary.pdf]

Figure S1: the stratified VPC

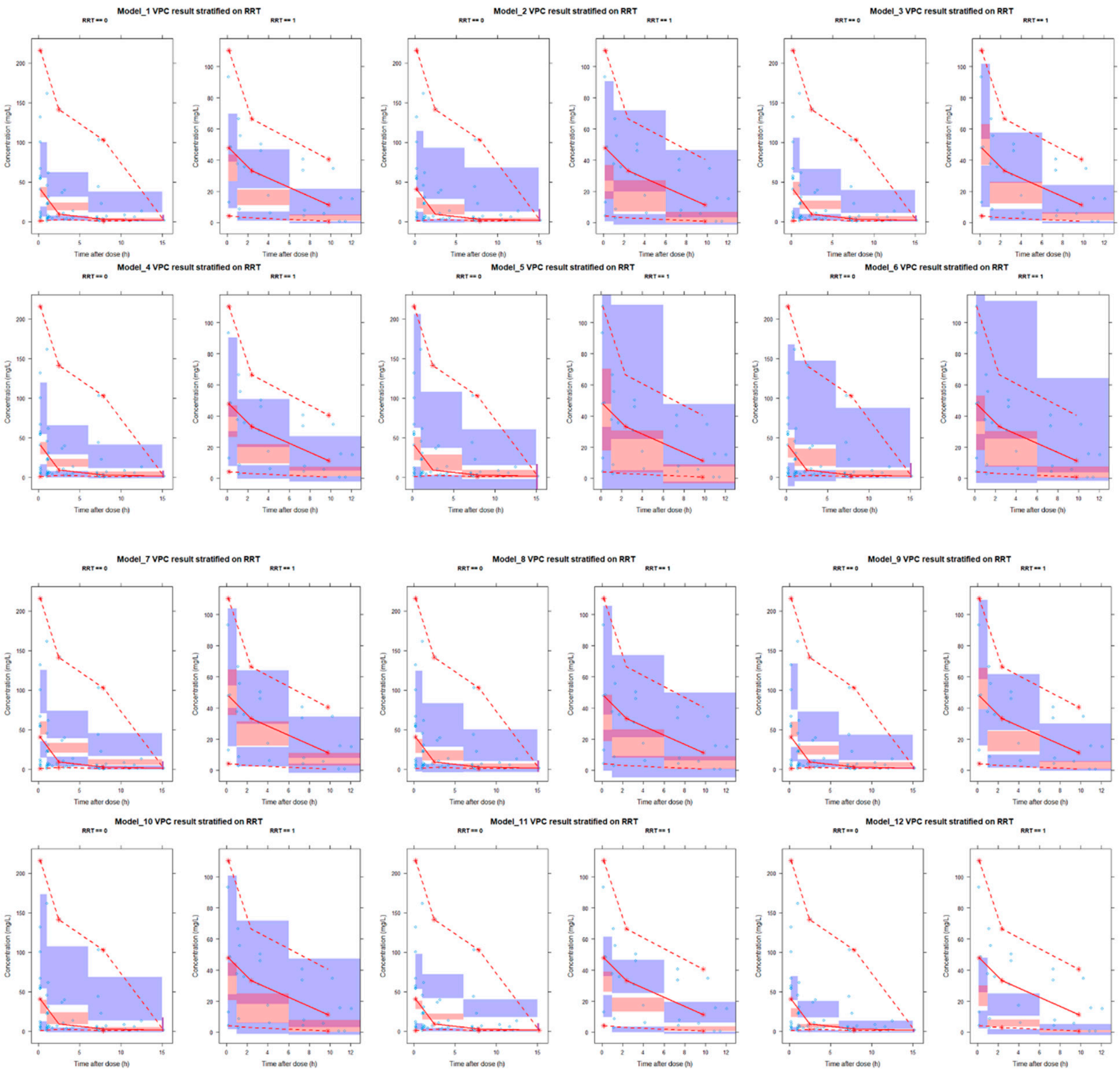

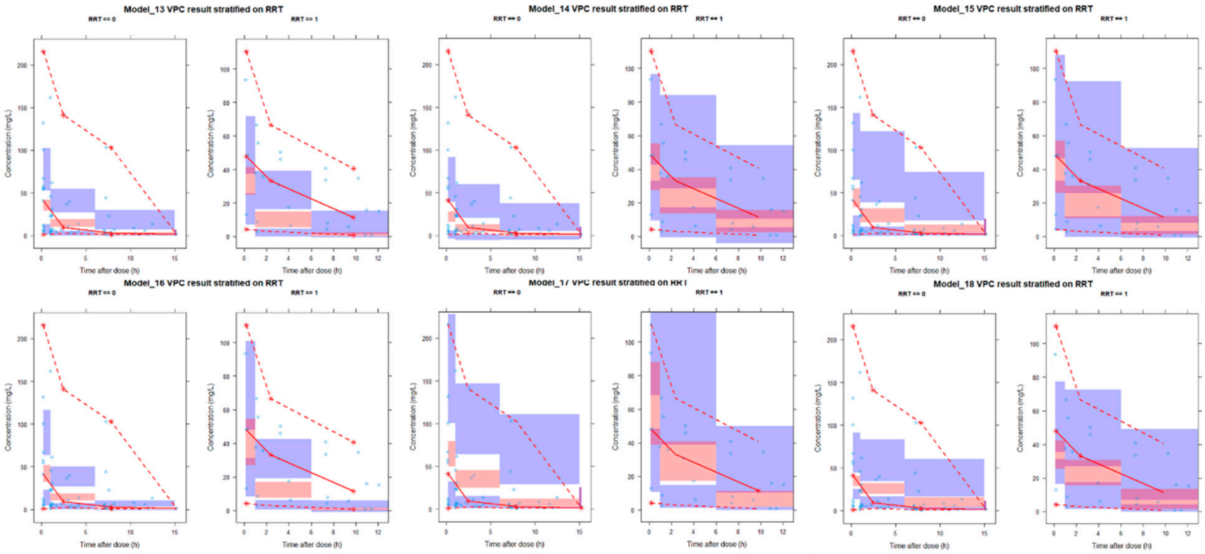

Stratified VPC results were based on patients received renal replacement therapy (RRT) or not. The bins were divided based on the peak (time after dose 0-1 hour), trough (time after dose 6-15 hour) and in between (time after dose 1-6 hour)

Figure S2: Population prediction errors of the models

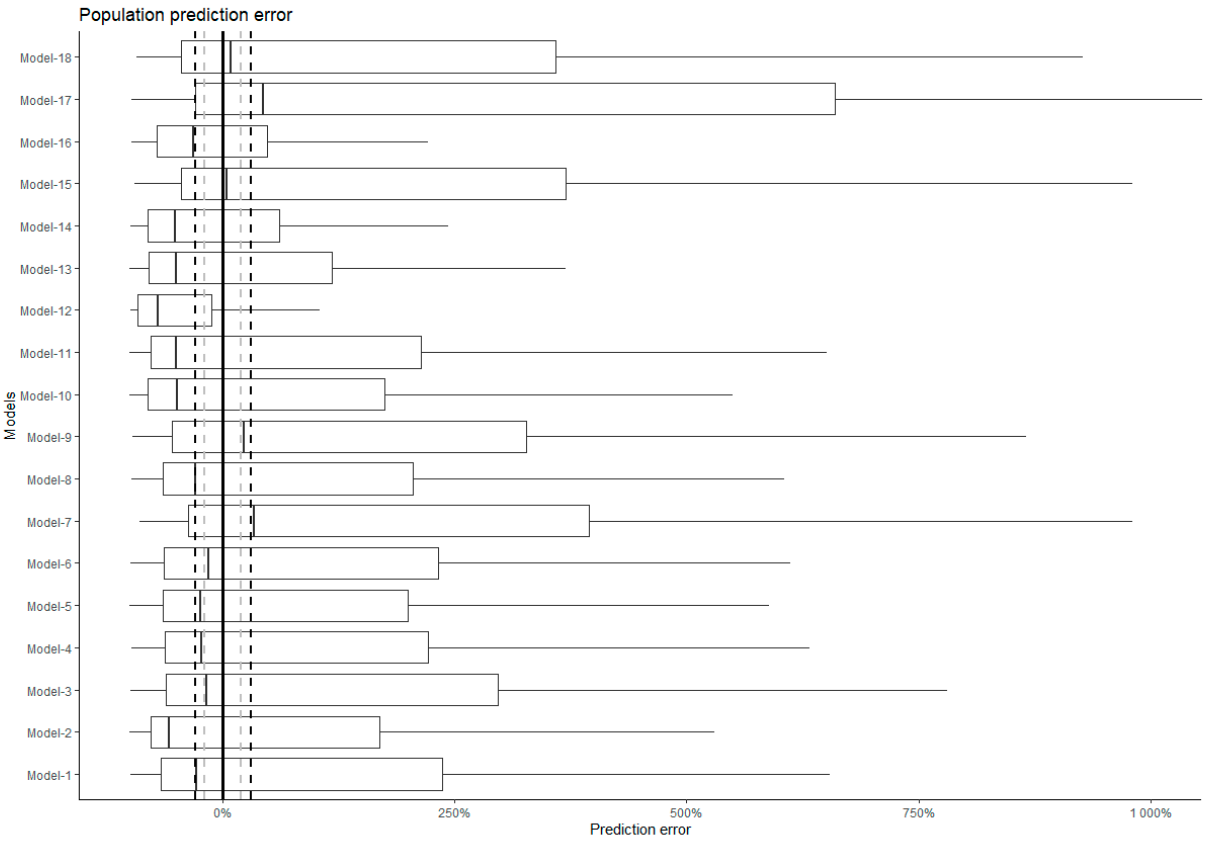

Summary of the population prediction error (PE) of the 18 models, the black dashed line is -30% and 30%, the grey dashed line is -20% and 20%. PE%=(predict-observed)/observed *100%

Figure S3 Individual prediction errors of the models

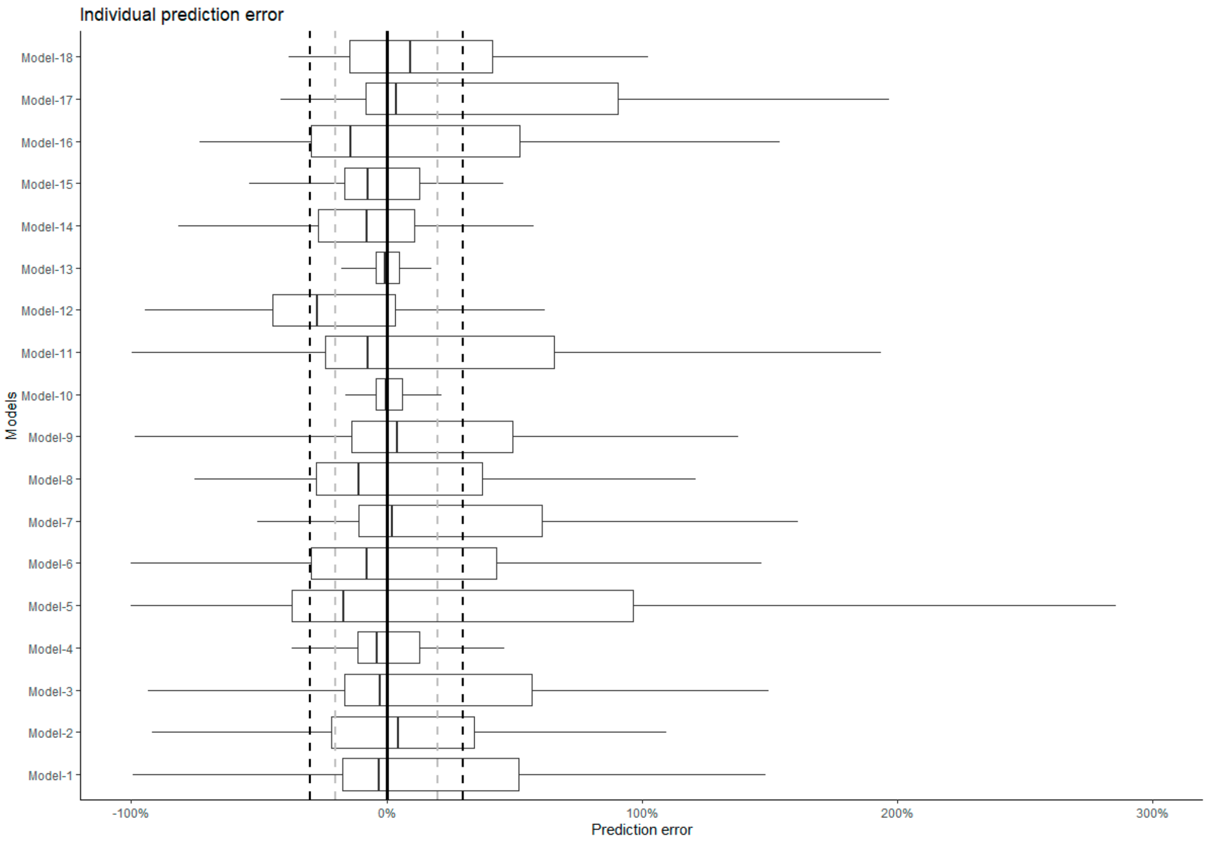

Summary of the individual prediction error (PE) of the 18 models, the black dashed line is -30% and 30%, the grey dashed line is -20% and 20%. PE%=(predict-observed)/observed *100%

Figure S4: Individual prediction errors after the models included one trough concentration

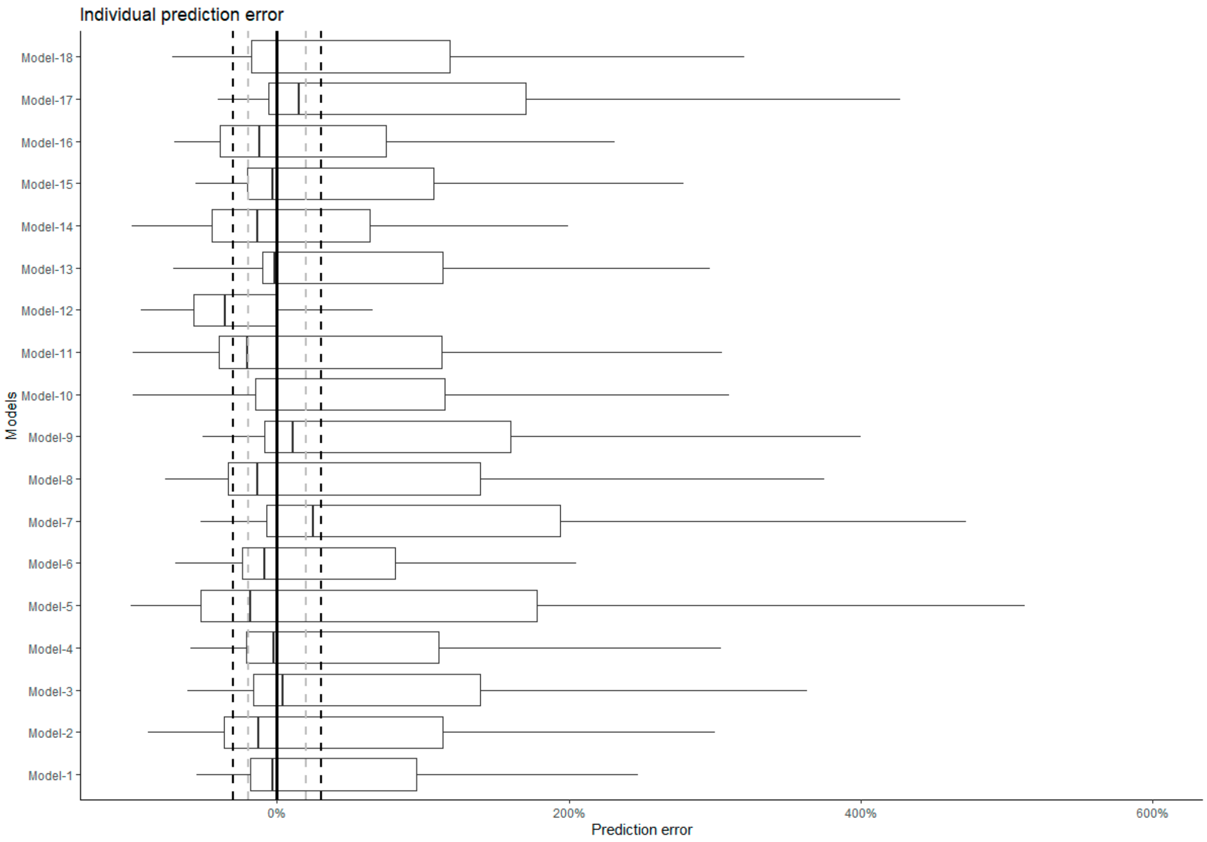

Summary of the individual prediction error (PE) with 1 concentration (trough concentration) incorporated into the model, PE%=(predict-observed)/observed *100%

Figure S5: Individual prediction errors after the models included two concentrations (trough and peak)

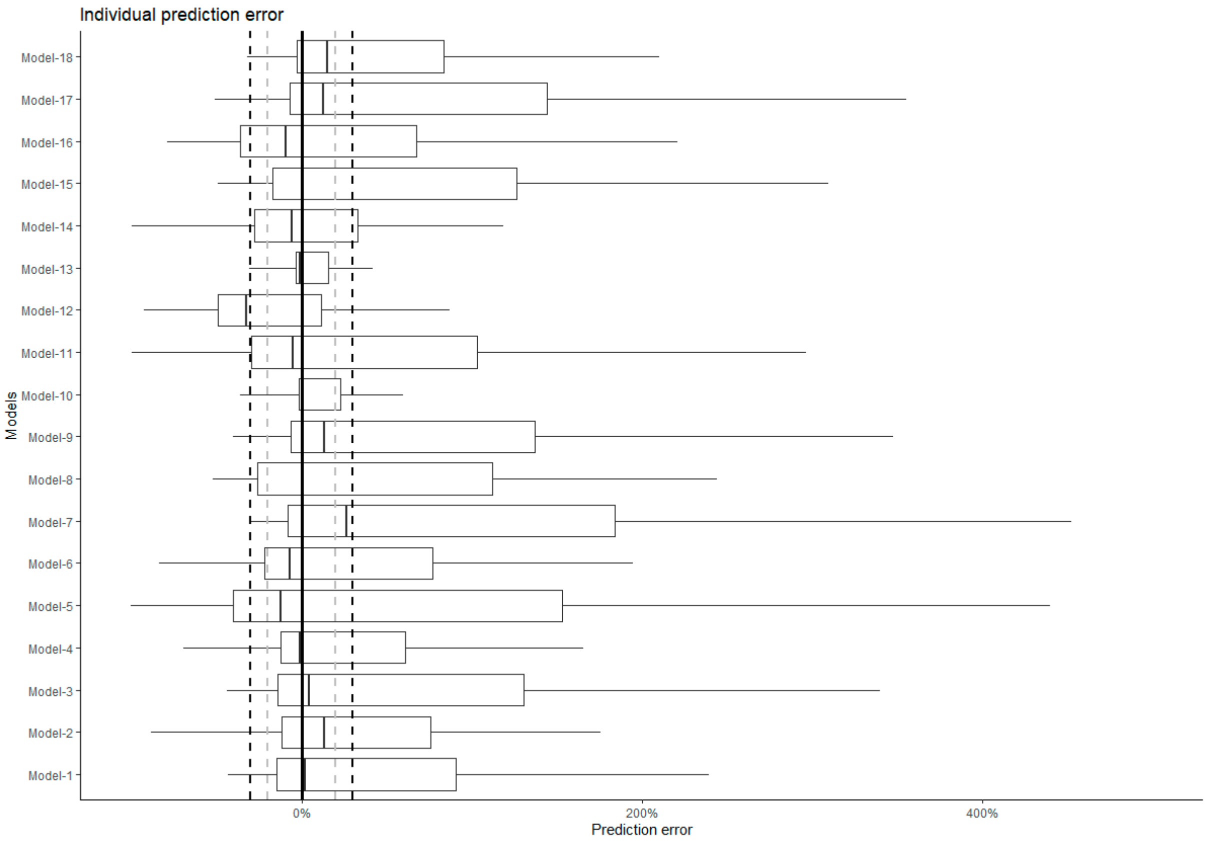

Summary of the individual prediction error (PE) with 2 concentration (trough concentration) incorporated into the model, PE%=(predict-observed)/observed *100%

Figure S6: Individual prediction errors after the models included three concentrations (trough and peak and in between)

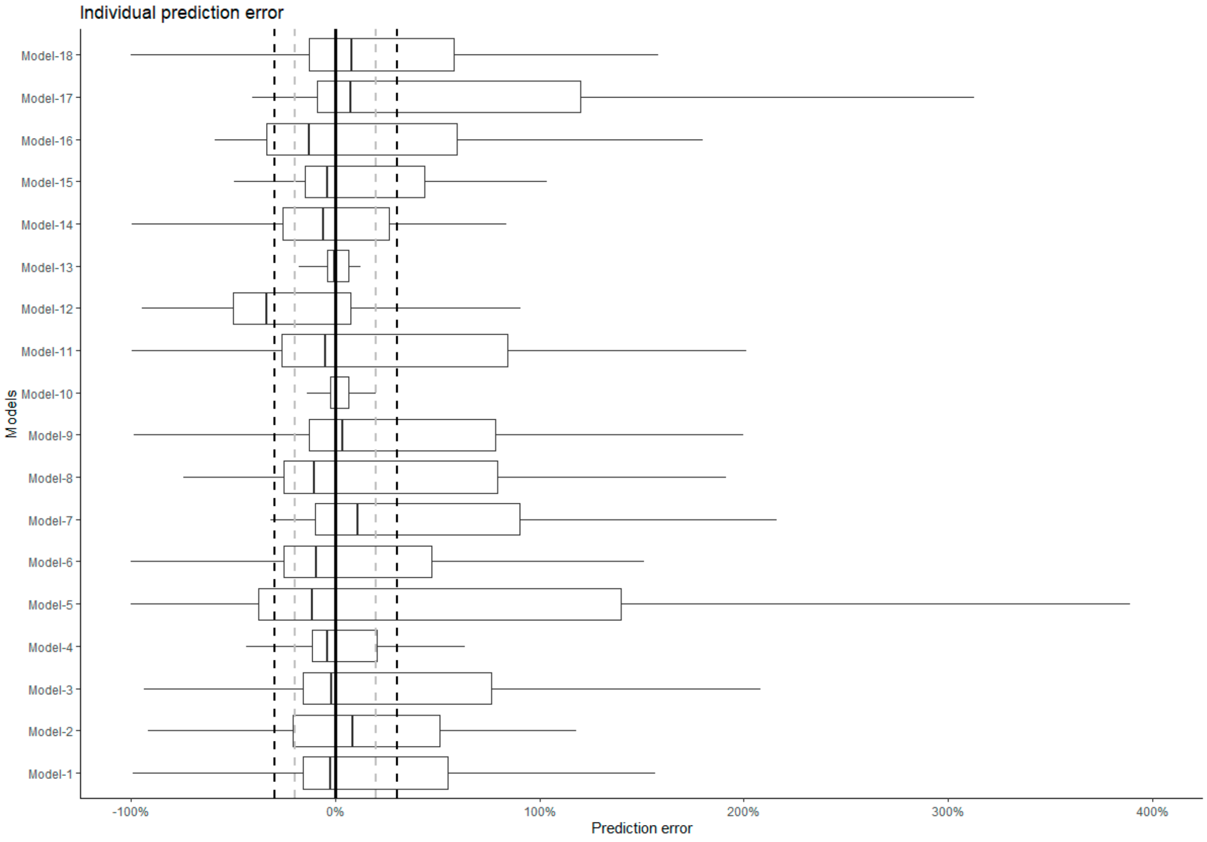

Summary of the individual prediction error (PE) with 3 concentration (trough concentration) incorporated into the model, PE%=(predict-observed)/observed *100%)

Figure S7: The RMSE of the models under different number of concentrations, Text S8: Literature search strategy

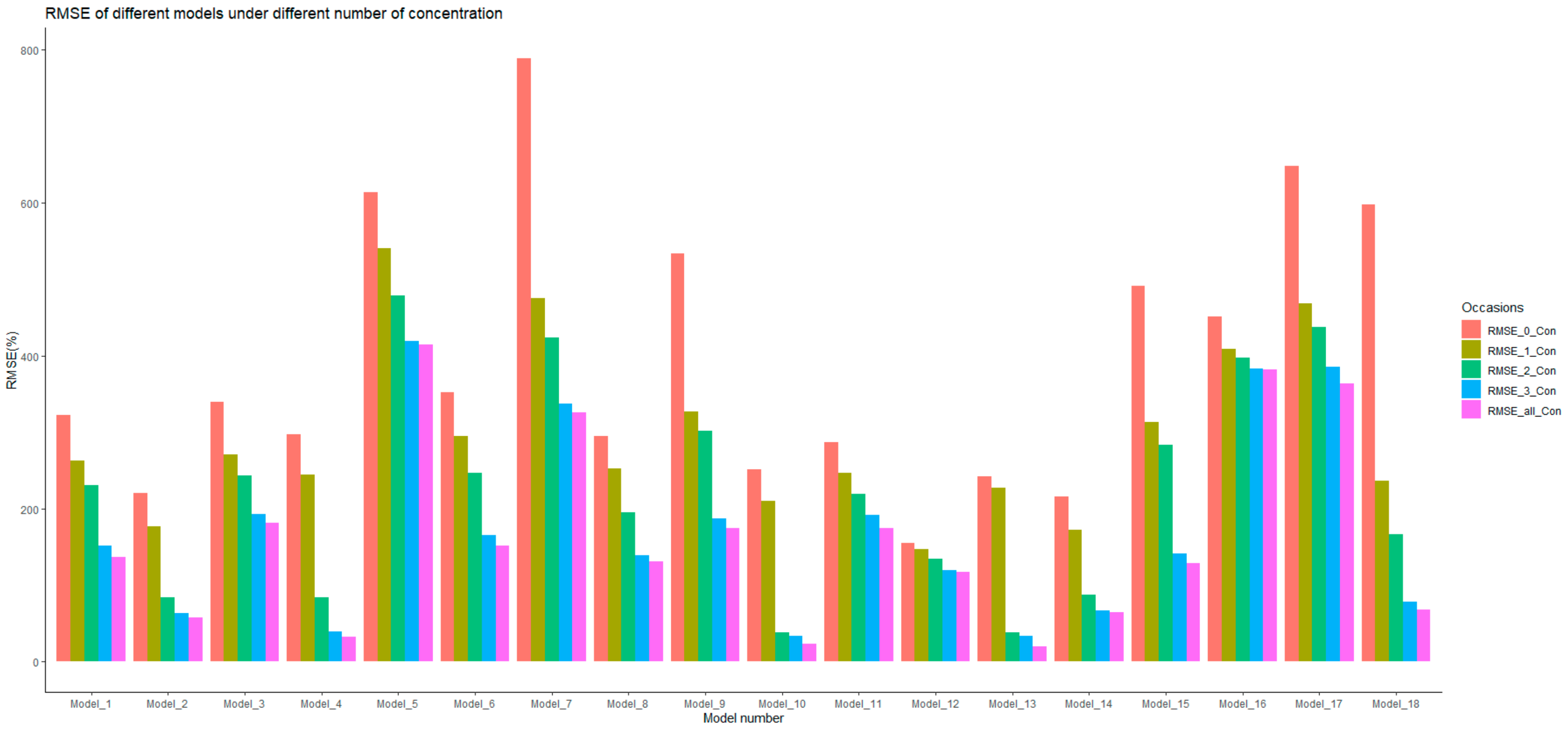

Summary of the root mean squared relative prediction error (RMSE) with each model under different concentration number included (RMSE_0_Con means no prior concentration incorporated into the models, RMSE_1_Con means 1 concentration incorporated into the models, RMSE_2_Con means 2 concentrations incorporated into the models, RMSE_3_Con means 3 concentrations incorporated into the models and RMSE_all_Con means all the patient concentrations incorporated into the models)

Text S1: Literature search strategy

Pubmed: (meropenem[mh] OR meropenem*[tiab] OR Merrem*[tiab] OR Ronem*[tiab] OR Penem*[tiab] OR SM-7338[tiab] OR SM7338[tiab]) AND (NONMEM OR nonlinear-Mixed-Effect* OR mixed-effect-model* OR non-linear-mix* OR Monolix*)

Embase: (('meropenem'/exp OR meropenem:ti,ab OR mepem:ti,ab OR meronem:ti,ab OR meropen:ti,ab OR merrem:ti,ab OR sm7338:ti,ab) AND (nonmem OR 'nonlinear mixed effects modeling' OR Monolix))
